# Using single nucleotide polymorphism array for prenatal diagnosis in a large multicenter study in Southern China

**DOI:** 10.1038/s41598-023-33668-0

**Published:** 2023-05-04

**Authors:** Meiying Cai, Na Lin, Nan Guo, Linjuan Su, Xiaoqing Wu, Xiaorui Xie, Ying Li, Shuqiong He, Xianguo Fu, Liangpu Xu, Hailong Huang

**Affiliations:** 1 Medical Genetic Diagnosis and Therapy Center, Fujian Maternity and Child Health Hospital College of Clinical Medicine for Obstetrics & Gynecology and Pediatrics, Fujian Medical University, Fujian Key Laboratory for Prenatal Diagnosis and Birth Defect, Fuzhou, China; 2 Department of Prenatal Diagnosis, Ningde Municipal Hospital, Ningde Normal University, Ningde, China

**Keywords:** Biological techniques, Genetics, Molecular biology, Health care, Medical research, Molecular medicine

## Abstract

Numerous studies have evaluated the use of single nucleotide polymorphism array (SNP-array) in prenatal diagnostics, but very few have evaluated its application under different risk conditions. Here, SNP-array was used for the retrospective analysis of 8386 pregnancies and the cases were categorized into seven groups. Pathogenic copy number variations (pCNVs) were found in 699 (8.3%, 699/8386) cases. Among the seven different risk factor groups, the non-invasive prenatal testing-positive group had the highest pCNVs rate (35.3%), followed by the abnormal ultrasound structure group (12.8%), and then the chromosomal abnormalities in the couples group (9.5%). Notably the adverse pregnancy history group presented with the lowest pCNVs rate (2.8%). Further evaluation of the 1495 cases with ultrasound abnormalities revealed that the highest pCNV rates were recorded in those cases with multiple system structure abnormalities (22.6%), followed by the groups with skeletal system (11.6%) and urinary system abnormalities (11.2%). A total of 3424 fetuses with ultrasonic soft markers were classified as having one, two, or three ultrasonic soft markers. The different pCNV rates in the three groups were statistically significant. There was little correlation between pCNVs and a previous history of adverse pregnancy outcomes, suggesting that genetic screening under these conditions should be evaluated on a case-by-case basis.

## Introduction

Methods of chromosome evaluation using amniotic fluid, umbilical cord blood, and chorionic villus sampling have been widely used since the 1970s. These methods have served as the gold standard for the diagnosis of fetal abnormalities^1^, especially in pregnant women with fetal malformations identified by ultrasound imaging or those with higher risk second trimester serum screening (STSS) results. Currently, the criteria to perform invasive prenatal diagnostic tests are (1) non-invasive prenatal testing (NIPT)-positive results; (2) abnormal ultrasound structures; (3) chromosomal abnormalities in couples; (4) high-risk STSS results; (5) advanced maternal age (≥ 35 years); (6) ultrasound soft markers; and (7) adverse pregnancy history. However, karyotype analysis cannot identify copy number variations (CNVs) of less than 10 Mb^2^, requires technical expertise and is subject to a long detection cycle^3^. Single nucleotide polymorphism array (SNP-array) is one of the many techniques used in chromosome microarray analysis (CMA). These arrays facilitate high resolution, high-throughput whole genome screening and SNP-array detection of CNVs that cannot be detected by karyotype analysis, including chromosomal microdeletion or microduplication. Uniparental disomy, polyploidy, and low percentage chimeras can also be detected using these techniques^4–7^.

Many studies have proposed CMA as a first-line prenatal diagnostic method^1,6^, especially in women with high-risk pregnancies, as it provides faster but effective prenatal diagnosis. Furthermore, numerous studies have evaluated the application of SNP-array in prenatal diagnosis^8–13^, but relatively few have evaluated performance differences in response to different risk factors. Here, we present a retrospective evaluation of SNP-array data obtained from 8386 fetuses using risk factor stratification to explore the performance of SNP-array-mediated evaluation with different risk factors, and the etiological relationship between these risk factors and CNV. These data could help inform the selection of appropriate prenatal diagnostic techniques for patients with different risk factors.

## Methods

### Ethics declaration

These experiments were approved by the ethics committee at the Fujian Provincial Maternal and Children Health Hospital (2014-042) and written-informed consent was obtained from all participants. All experiments were performed in accordance with relevant guidelines and regulations.

### Study subjects

We performed a retrospective analysis of SNP-array data obtained from the evaluation of 8386 fetuses who underwent prenatal diagnosis at Fujian Maternity and Child Health Hospital, a tertiary referral center in Southern China, between January 2016 and November 2021. Each of the pregnant women signed the informed consent form and underwent interventional prenatal diagnostic puncture, as approved by the Ethics Committee at Fujian Maternal and Child Health Hospital. The ages of the pregnant women ranged from 18 to 48 years, and the gestational ages of the fetuses ranged from 11 to 36 weeks. Villi, amniotic fluid,and umbilical cord blood were all collected according to the gestational age guidelines outlined for SNP-array detection of CNV. Villocentesis was performed at 11–13 weeks of gestation, amniocentesis was performed at 17–24 weeks of gestation, and cord blood biopsy was performed at 25–36 weeks of gestation. Of the total 8386 samples obtained, 0.7% (62/8386) were villi samples, 83.1% (6970/8386) were amniotic fluid samples, and 16.1% (1354/8386) were cord blood samples. We also split the 8386 samples into seven groups to facilitate risk evaluation as follows: (1) NIPT positive (*n* = 323); (2) abnormal ultrasound structure (*n* = 1495); (3) chromosomal abnormalities in couples (*n* = 232); (4) high-risk STSS (*n* = 1100); (5) advanced maternal age (≥ 35 years; *n* = 1176); (6) ultrasound soft markers (*n* = 3423); and (7) adverse pregnancy history (*n* = 637).

### DNA extraction

Villus, umbilical cord, and amniotic fluid DNA was extracted using a genomic DNA extraction kit (QIAGEN, Germany) as described by the manufacturer. The extracted DNA was stored at −20 °C for future use.

### SNP-array detection and data analysis

We used a high-resolution whole-genome CytoScan 750 K chip from Affymetrix to facilitate SNP-array typing, with DNA digestion, amplification, purification, fragmentation, labeling, hybridization, staining, and scanning performed as described by Affymetrix. The results of our GRCH37 (Human Genome 19, HG19) analyses were then compared using Chromosome Analysis Suite software, and the results were interpreted using the reference databases DGV (http://projects.tcag.ca/variation), DECIPHER (https://www.deciphergenomics.org/), Clinvar (http://www.Clinicalgenome.org/data-sharing/Clinvar/), PubMed, and OMIM (www.ncbi.nlm.nih.gov/OMIM). We then used the clinical significance guidelines for CNV developed by the American College of Medical Genetics and Genomics^14,15^ to divide our results into three categories and five levels: (1) pathogenic CNV (pCNV) (including pathogenic CNV and likely pathogenic CNV), (2) benign CNV(including benign CNV and likely benign CNV), and (3) variants of unknown significance (VUS). The medium length of time needed to perform SNP-array was 10 days.

### Statistical analysis

Statistical analyses were performed using SPSS 22.0 (IBM, Armonk, NY). All data are expressed as rates, and a χ2 test was used to compare values between different groups. Statistical significance was set at *p* < 0.05.

## Results

### SNP-array

SNP-array analysis of all 8386 samples was successfully conducted, with 7553 samples (90.1%, 7553/8386) returning a normal CNV result and only 833 samples (9.9%, 833/8386) returning an abnormal CNV result. Of the 833 CNV abnormalities, 699 were pCNV and 134 were VUS. Within the pCNV group, we identified 266 cases of aneuploidy, of which 110 were trisomy 21-syndrome, 47 were XXY syndrome, and 40 were trisomy 18-syndrome. The most common microdeletion/microduplication syndrome in the pCNV group was 22q11.21 microdeletion syndrome (33 cases), followed by 16p11.2 microdeletion syndrome (19 cases), and 17q12 microdeletion syndrome (eight cases).

### Relationship between different risk factors and CNV

The abnormal CNV rate for each of the seven groups with high-risk factorsis shown in Table 1. The pCNV rate in the NIPT-positive group was 35.3% (114/323), whereas it was only 12.8% (192/1495) in the abnormal ultrasound structure group, 9.5% (22/232) in couples with chromosomal abnormalities, 8.0% (88/1100) in the high-risk STSS group, 5.8%in both the advanced age (68/1176) and ultrasound soft markers groups (197/3423), and 2.8% (18/637) in the adverse pregnancy history group. The difference in these pCNV rates was statistically significant (*p* < 0.05; Table 1).

**Table 1:** Different risk factors and CNV.

Risk factors	Total cases	Total abnormal number of CNV	Number of pCNV	Number of VUS
NIPT-positive	323	121	114	7
Abnormal ultrasound structure	1495	226	192	34
Chromosomal abnormalities in couples	232	25	22	3
STSS high-risk	1100	100	88	12
Advanced age	1176	86	68	18
Ultrasonic soft marker	3423	243	197	46
Adverse pregnancy history	637	32	18	14

Abbreviations: *NIPT* non-invasive prenatal testing, *STSS* second trimester serum screening, *pCNV* pathogenic copy number variation, *VUS* variants of unknown significance.

### Abnormal ultrasonic structure categories and CNVs

A total of 1495 fetuses with abnormal ultrasound structures underwent SNP-array (Table 2). The severity and type of abnormalities were evaluated and classified based on the anatomical system in which the structural abnormalities occurred, and the number of systems affected. This stratification resulted in eight groups, and the pCNV rate was highest inthe multiple system structures group (22.6%, 57/252), followed by the skeletal system (11.6%, 11/95), urinary system (11.2%, 30/268), nervous system (11.2%, 19/170), cardiovascular system (7.6%, 61/807), respiratory system (7.3%, 4/55), digestive system (6.9%, 6/87), and facial abnormality groups (5.4%, 4/74).

**Table 2:** Types of ultrasonic structure and CNV.

Types of ultrasonic structure	Total cases	Abnormal number of CNV	Percentage of abnormal CNV (%)	Number of pCNV	Number of VUS
Skeletal system	95	13	13.7	11	2
Nervous system	170	23	13.5	19	4
Urinary system	268	34	12.7	30	4
Facial abnormality	74	8	10.8	4	4
Respiratory system	55	5	9.1	4	1
Cardiovascular system	807	73	9.0	61	12
Digestive system	87	7	8.0	6	1
Multiple system	252	63	25.0	57	6

Abbreviations: *pCNV* pathogenic copy number variation, *VUS* variants of unknown significance.

### Ultrasound soft markers and CNV

Our ultrasound evaluations identified 3424 cases of ultrasound soft markers, which were then divided into three groups based on the number of abnormalities (one, two, or ≥ three) reported in each case (Table 3). We then compared the pCNV rates in these groups and found that the ≥ three ultrasound soft markers group had the highest pCNV rate (11.3%, 45/397), followed by the two ultrasound soft markers group (5.8%, 63/1078), and then the one ultrasound soft markers group (4.6%, 89/1949). These pCNV rates were statistically significantly different (*p* < 0.05).

**Table 3:** Number of ultrasonic soft marker and CNV.

Number of ultrasonic soft marker	Total cases	Total abnormal number of CNV	Number of pCNV	Number of VUS
1 ultrasonic soft marker group	1949	117	89	28
2 ultrasonic soft markers group	1078	77	63	14
≥ 3ultrasonic soft markers group	397	49	45	4

Abbreviations: *pCNV* pathogenic copy number variation, *VUS* variants of unknown significance.

### Pregnancy outcomes

All subjects underwent telephone or outpatient follow-up, and the records indicate that 7547 of the 8386 cases (90%) were successfully followed up. Of the 699 fetuses with pCNVs, 560 were terminated by the parents and 16 fetuses were allowed to continue, producing 11 healthy babies and five infants with abnormal growth or development (Table 4). The remaining 23 cases were lost during follow-up. Of the 134 fetuses with VUS, only 16 cases were terminated, and the remaining 103 cases resulted in 99 healthy infants and four cases of abnormal growth and development (Table 5). Only 15 of these cases were lost to follow-up.

**Table 4:** The postnatal outcomes of 16 fetuses with pCNVs.

Case	Risk factors	SNP-array	Postnatal outcome
1	Ultrasonic soft marker	arr[hg19]16p13.11(15,510,512–16,309,046) × 3	Normal
2	Ultrasonic soft marker	arr[hg19]16p13.11(15,058,820–16,309,046) × 3	Normal
3	STSS high-risk	arr[hg19]16p13.11(14,920,864–16,538,596) × 3	Normal
4	Chromosomal abnormalities in couples	arr[hg19]16p13.11(14,929,070–16,272,403) × 3	Normal
5	Ultrasonic soft marker	arr[hg19]16p13.11(15,481,747–16,272,403) × 3	Normal
6	Advanced age	arr[hg19]16p13.11p12.3(15,325,072–18,242,713) × 3	Normal
7	Ultrasonic soft marker	arr[hg19]16p13.11(15,058,820–16,538,596) × 3	Normal
8	Ultrasonic soft marker	arr[hg19]1q21.1q21.2(146,525,270_147,391,923) × 3	Normal
9	Advanced age	arr[hg19]1q21.1q21.2(145,124,436_147,995,251) × 3	Normal
10	STSS high-risk	arr[hg19]1q21.1q21.2(146,106,723_147,933,973) × 1	Normal
11	Ultrasonic soft marker	arr[hg19] Xp22.33 or Yp11.32(168,551–629,999 or 118,551–579,999) × 1	Normal
12	Ultrasonic soft marker	arr[hg19] Xp22.33 or Yp11.32(803,294–1,519,822 or 753,294–1,469,822) × 1	Abnormal growth or development
13	Ultrasonic soft marker	arr[hg19]17q21.31(41,74,473–42,491,805) × 4	Abnormal growth or development
14	Ultrasonic soft marker	arr[hg19] 22q11.21(21058887_21464764) × 1	Abnormal growth or development
15	Ultrasonic soft marker	arr[hg19]1q21.1q21.2(146,096,700_147,391,923) × 3	Died 2 days after birth
16	Abnormal ultrasound structure	arr[hg19] 22q11.21(21058887_21464764) × 1	Died after surgery of ventricular septal defect

**Table 5:** The postnatal outcomes of 4 fetuses with VUS.

Case	Risk factors	SNP-array	Postnatal outcome
1	Ultrasonic soft marker	arr[hg19] 18p11.23p11.22(7,153,845–8,964,650) × 3	Abnormal growth or development
2	Ultrasonic soft marker	arr[hg19] 17q21.31(41,774,473–42,491,805) × 4	Abnormal growth or development
3	Advanced age	arr[hg19] 2p25.3p11.2(50,813–87,053,152) × 3	Abnormal growth or development
4	Ultrasonic soft marker	arr[hg19] 10q11.22q11.23(46,252,072–51,903,756) × 3	Abnormal growth or development

## Discussion

Although traditional chromosome karyotyping can identify polyploidy, aneuploidy, translocation, inversion, chimerism, duplication, and deletions larger than 10 Mb, this technique has various limitations, including time-consuming cell cultures, low resolution, high labor demand, and an inability to identify CNVs smaller than 10 Mb^11,16–19^. SNP-array can not only detect CNVs but also most cases of uniparental disomy, triploidy, and low percentage chimeras. Furthermore, it is suitable for the analysis of fresh, frozen, and formalin-fixed or paraffin-embedded tissues, making it a clear choice for first-line evaluation^2,20,21^.

Here, SNP-array was used to evaluate CNV abnormalities in 8386 fetuses presenting one or more risk factors for birth defects. These evaluations identified pCNV mutations in 8.3% (699/8386) of the cases. However, when we grouped these data using different risk factors, we noted that some risk factor groups presented significantly increased pCNV rates. The highest pCNV rate was 35.3% in the NIPT-positive group. The second highest pCNV rate (12.8%) was identified in the abnormal ultrasound structure group. This was consistent with the findings of Wapner et al.^9^ and Hillman et al.^22^. Risk factor stratification also revealed a reasonably high pCNV rate for couples with chromosomal abnormalities, reaching nearly 9.5% in some cases. This may be a result of chromosomal abnormalities increasing the risk of fetal pCNVs, which should be considered when performing counseling and evaluating screening outcomes. However, the small sample size of the couples with chromosomal abnormalities may have influenced these results; thus, this evaluation should be repeated using a larger cohort. Our data also revealed increased pCNV rates (8.0%) for pregnancies identified with high-risk STSS results. Although the pCNV rate for this group was lower than that of the NIPT-positive group, STSS identifies high-risk abnormalities in the neural tube, which NIPT cannot, therefore it cannot be replaced by NIPT. Instead, these results suggest STSS could be combined with NIPT to further improve screening accuracy for high-risk pregnancies. There has also been a significant increase in the number of pregnancies in older women in recent years, which has correlated with a significant increase in the risk of birth defects^23^. The pCNV rate in the advanced age group was 5.8%, which is similar to the 6.7% reported by Donnelly et al.^24^ and 3.0% reported by Fiorentino et al.^25^. However, our pCNV rate was significantly higher than the 1.52% reported by Zhu et al.^26^. The main reason for this difference may be that, in the current study, we used an SNP-array, whereas Zhu et al.^26^ used karyotype analysis. Finally, the pCNV rate was the lowest (2.8%) in patients with an adverse pregnancy history, suggesting that there was only a small overlap between genetic abnormality and miscarriage in this population. Considered together, these results indicate that genetic screening of patients with a history of adverse pregnancy outcomes, under these conditions, should be evaluated on a case-by-case basis.

Ultrasound scans are increasingly being used to detect structural abnormalities in the fetus during prenatal testing, and increased resolution has facilitated improved detection of minor abnormalities. Fetal ultrasound abnormalities caused by pCNV may be accompanied by cognitive dysfunction and developmental delay, and these abnormalities cannot be detected by ultrasound. Therefore, it is necessary to conduct genomic analysis in cases of ultrasound abnormalities to ensure a complete diagnostic picture.

The pCNV detection rates of specific CMA are different depending upon the fetal structural abnormalities, being more commonly reported in fetuses with physical abnormalities in the cardiovascular, nervous, skeletal, and urinary systems^24,27–30^. Our findings partially agree with this; of the 1495 fetuses with abnormal ultrasound structures, those with multi-system abnormalities presented the highest rates of pCNV (22.6%). However, the rates of pCNV in single system structure abnormalities were 11.6%, 11.2%, 11.2%, 7.6%, and 7.3% in the skeletal, urinary, nervous, cardiovascular, and respiratory systems, respectively. These results suggest that SNP-array should be recommended for pregnant women when ultrasound examination reveals structural abnormalities in multiple fetal systems, as well as in cases where there are clear skeletal, urinary, nervous, cardiovascular, or respiratory system abnormalities. However, the rates of pCNV for the digestive system (6.9%) and facial abnormalities (5.4%) remained reasonably low, suggesting that different prenatal diagnostic techniques should be selected accordingly.

Ultrasound soft markers are commonly found in fetuses with chromosomal abnormalities, including absence or dysplasia of the nasal bones, thickening of the neck skin, dilation of the lateral ventricles, bright spots in the heart, enhanced echo of the bowel, short limbs, mild dilation of the renal pelvis, and a single umbilical artery^31^. These nonspecific, minor fetal structural changes may be normal and disappear as pregnancy progresses, but they may also indicate increased fetal risk for pCNV. However, performing invasive prenatal diagnostic methods on the basis of these non-structural anomalies remains controversial. Here, we note that the rates of pCNV were 4.6%, 5.8%, and 11.3% in fetal groups with one, two, and ≥ three ultrasound soft markers. Thus, the rate of pCNV increased with an increase in the number of ultrasound soft markers. This suggests that an SNP-array should be performed on pregnant women when multiple ultrasound soft markers are detected by ultrasound. At present, the interpretation of many ultrasound soft markers still requires the evaluation and verification of a large amount of clinical data. Clinicians should consider ultrasound soft markers objectively and accurately based on existing data and improve interpretation of indicators or invasive prenatal diagnostic results, provide necessary genetic counseling and informed choices for pregnant women, and avoid exaggerating or ignoring the role of ultrasound soft markers in high-risk pregnancies.

Given the powerful diagnostic capacity of SNP-array for pCNV, many VUS with unclear phenotypic relevance have been detected^32,33^. The presence of VUS in prenatal diagnosis often leads to difficulties in genetic counseling, stress on the pregnant woman and her family, and excessive termination rates. VUS account for less than 5% of all cases^34^, and our data identified only 134 cases with likely VUS abnormalities out of the 8386 cases, a detection rate of 1.6%, which is consistent with the data reported in previous literature. Methods for the accurate evaluation of VUS cases needs to be further determined by clinicians. VUS cases are the most likely to have favorable pregnancy outcomes, therefore, cases presenting with VUS abnormalities require further follow-up. This could be important in informing the development of guidelines for genetic counseling in the future.

Our study had some shortcomings. First, we only used SNP-array data. There are still some problems associated with the clinical application of SNP-array, including the inability to detect real chromosomal equilibrium aberrations and difficulties in interpreting the pathogenicity of a large number of tiny fragments. Second, follow-up of the fetus after birth could have been improved, especially for fetuses with abnormal ultrasound structures, but normal VUS as there was no continuous tracking of information, and other possible causes of abnormal ultrasound structure were not considered. Third, fetuses with VUS were not tested further using other technical approaches, such as next-generation sequencingy^35–40^. In future studies, these deficiencies can be improved to facilitate better detection of fetal abnormalities and to better inform appropriate clinical interventions.

## Conclusions

We found that there was little correlation between pCNV and a previous history of adverse pregnancy outcomes, suggesting that genetic screening under these conditions should be evaluated on a case-by-case basis. In the same way, different prenatal diagnostic techniques should be selected for fetuses with digestive system abnormalities, facial abnormalities, and when only one ultrasound soft marker is detected by ultrasound.

## Data Availability

The datasets generated and/or analyzed during the current study are available from the corresponding author.
